# Identifying socio-demographic risk factors for suicide using data on an individual level

**DOI:** 10.1186/s12889-021-11743-3

**Published:** 2021-09-18

**Authors:** Guus Berkelmans, Rob van der Mei, Sandjai Bhulai, Renske Gilissen

**Affiliations:** 1 Centrum Wiskunde & Informatica, Science Park 123, 1098 XG Amsterdam, Netherlands; 2 Vrije Universiteit Amsterdam, De Boelelaan 1111, 1081 HV Amsterdam, Netherlands; 3 113 zelfmoordpreventie, Paasheuvelweg 25, 1105 BP Amsterdam, Netherlands

**Keywords:** Suicide, Machine learning, Big data, Risk factors

## Abstract

**Background:**

Suicide is a complex issue. Due to the relative rarity of the event, studies into risk factors are regularly limited by sample size or biased samples. The aims of the study were to find risk factors for suicide that are robust to intercorrelation, and which were based on a large and unbiased sample.

**Methods:**

Using a training set of 5854 suicides and 596,416 control cases, we fit a logistic regression model and then evaluate the performance on a test set of 1425 suicides and 594,893 control cases. The data used was micro-data of Statistics Netherlands (CBS) with data on each inhabitant of the Netherlands.

**Results:**

Taking the effect of possible correlating risk factors into account, those with a higher risk for suicide are men, middle-aged people, people with low income, those living alone, the unemployed, and those with mental or physical health problems. People with a lower risk are the highly educated, those with a non-western immigration background, and those living with a partner.

**Conclusion:**

We confirmed previously known risk factors such as male gender, middle-age, and low income and found that they are risk factors that are robust to intercorrelation. We found that debt and urbanicity were mostly insignificant and found that the regional differences found in raw frequencies are mostly explained away after correction of correlating risk factors, indicating that these differences were primarily caused due to the differences in the demographic makeup of the regions. We found an AUC of 0.77, which is high for a model predicting suicide death and comparable to the performance of deep learning models but with the benefit of remaining explainable.

## Introduction

Suicide is a complex issue that involves multiple factors. Many researchers have looked into risk factors for suicide. However, much of this research looks at risk factors in isolation, or corrected only for age or gender [1–5]. As a consequence, risk factors found in these studies could simply be a proxy for other risk factors due to the fact that they are correlated (for example, education level and income). Additionally, many studies are of limited size, and are usually non-representative of the population as a whole due to the way the selection procedure was set up, for example, a clinical setting [1].

Knowing that suicide is rarely related to just one risk factor, this study quantifies the effect of individual characteristics as accurately as possible by correcting for correlation of characteristics. Furthermore, this study uses all suicide cases in the Netherlands (around 1900 suicides are reported every year) and a large randomly selected sample of control cases drawn from the full population. This avoids issues of small sample size and selection bias.

To our knowledge, only Gradus et al. [6] used such an approach before in Denmark. They found sex-specific risk profiles for suicide, focusing their risk profiles mainly on medical data. However, in this paper, we focus on socio-demographic risk factors.

This study decorrelates the effects of the risk factors to obtain odds ratios which take into account the proxy effects to the other risk factors. Moreover, we look across multiple years (2014–2017) and at a large number of socio-demographic factors. In this way, we obtain risk factors that are both robust to intercorrelation as well as to events that raise the risk among a certain subpopulation.

## Methods

The primary aim of the study is to find risk factors for suicide that are robust to intercorrelation. In this way we can be sure that the risk factors are not proxies for the numerous other risk factors that are included in the study. Additionally, a secondary aim is to make sure that we can be sure that the risk factors found are based on a large unbiased sample.

The data used was the micro-data of Statistics Netherlands [7]. Statistics Netherlands collects data on each inhabitant of the Netherlands (approximately 17,000,000 inhabitants) from various sources, which are required to provide this information by law. This data includes socio-demographic characteristics like birth date, gender, marital status, type of household, role in household, ethnicity, income, social benefits and in case of death it includes cause and date of death.

Due to the privacy-sensitive nature of the data, it is not freely accessible, nor is the data itself allowed to be published. Access has to be granted by Statistics Netherlands on a project-to-project basis, which was granted for this project. It is only possible to work with the data via remote connection to their secure servers, and any output is checked on whether it satisfies the privacy regulations before it is released for publication.

We limited ourselves to the period of 2014–2017 since some of the databases for 2018 and later were still undergoing data quality checks. Additionally, some databases had a different format prior to 2014 so did not include all of the characteristics of interest prior to 2014. Therefore, we could not analyze data from before 2014 alongside data from the period 2014–2017 while retaining all characteristics of interest. From the dataset of the years 2014–2017, those individuals who died by suicide were identified based on their cause of death, as established by coroners (ICD-10 codes for external causes: intentional self-harm (X60-X84)). The coroner is contacted when there is doubt as to whether a person died of natural causes. The coroner is always contacted when the deceased is underage (in the Netherlands, this means younger than 18 years old).

### Statistical analysis

The binomial logit model was used (commonly referred to as logistic regression) to decorrelate effects. Socio-demographic characteristics of each inhabitant aged 10 and up on the 31st of December (of 2013, 2014, 2015, or 2016) were categorised. We limited ourselves to ages 10 and up since Statistics Netherlands doesn’t report on suicides among youths under 10 years old, due to it being an extremely rare event. We then modelled the probability of suicide according to a binomial logit model such that

$$ P\left({S}_n|\overrightarrow{x_n}\right)=\frac{e^{V\left(\overrightarrow{x_n}\right)}}{1+{e}^{V\left(\overrightarrow{x_n}\right)}}, $$

where *S*_*n*_ is the event that individual *n* dies due to suicide in the following year, and

$$ V\left(\overrightarrow{x_n}\right)={\beta}_0+\sum \limits_{j=1}^k{\beta}_j{\left(\overrightarrow{x_n}\right)}_j $$

where ${\left(\overrightarrow{x_n}\right)}_j$ is 1 if individual *n* has characteristic *j* and 0 otherwise, and *k* is the total number of possible characteristics. This results in characteristics *j* having an odds ratio (OR) of ${e}^{\beta_j}$.

Since suicide is a quite rare event (roughly 1 per 10,000 people per year), the odds which are defined as O $=\frac{p}{1-p}$ are extremely close to the actual probability.

The main advantage of such a model is that proxy effects are corrected for as long as the proxy is also included in the model. Therefore, risk groups that are heavily correlated with, e.g., age, gender, income are corrected for. Though there is still an underlying assumption that risk factors increase risk independently to a certain degree, this assumption is significantly weaker than if one considered the risk factors in isolation or if corrected for a small number of risk factors.

Estimation was done using the Python package *biogeme* [8]. This package estimates the model parameters using maximum likelihood estimation by gradient descent. It has been proven [9] that in the case of the binomial logit model, this always converges to the optimal model with regards to the training error. This means we do not have to worry about local optima. Additionally, the package provides us with standard errors on the parameter estimation, allowing us to form confidence intervals and do tests of significance. The tests of significance done are t-tests (which show how many standard deviations of the estimator it is distanced from 0).

First, estimation was done on a training set. This training set consisted of both people who died by suicide as well as a group of people who did not die by suicide. The people who died by suicide were included with independent probability 0.8 (ended up being 5854 cases). The people who did not die due to suicide were included with independent probability 0.01 (ended up being 596,416 cases). Due to the way the sampling was done, all bias introduced is introduced into the *β*_0_ parameter. We, therefore, do not report this parameter. The selection procedure of the training set does not introduce any bias into the other parameters.

Secondly, we generated a test set. This test set contained the remaining suicide cases (1425 cases). Additionally, it contained cases of people who did not die by suicide. These cases were again included with probability 0.01, in such a way that it contains no cases included in the training set.

We then estimated the predicted risk of suicide for this test set. From these predictions, we calculated the sensitivity (the proportion of correctly classified cases among suicide victims) and specificity (the proportion of correctly classified cases among those who did not die due to suicide) for various risk thresholds. We then plotted the sensitivity and specificity against each other. In this way, we obtained the receiver operating characteristics curve (ROC curve). We then calculated the area under the ROC curve (AUC) to estimate model performance. The AUC is also the probability that a random case of death by suicide gets a higher predicted risk than a random case of someone who does not die due to suicide.

## Results

The parameters we estimated (i.e., the *β*_*j*_ parameters and associated standard errors, t-tests, and odds-ratios) for the binomial logit model are shown in Table 1. When we talk about increased risk we are talking about increases to the odds of suicide.

**Table 1:** Socio-demographic risk and protective factors for suicide in the Netherlands

Categories	Characteristics	Beta Parameters	Std. errors	t-tests	Odds-ratio	N(%) training set	N(%) suicides training set
Age	10 to 19	0	Fixed	Fixed	1	79,525(13%)	195(3%)
	20 to 29	0.95	0.10	9.40***	2.58	80,131(13%)	541(9%)
	30 to 39	1.39	0.11	12.52***	4.01	79,243(13%)	677(12%)
	40 to 49	1.74	0.11	15.82***	5.70	97,348(16%)	1159(20%)
	50 to 59	1.9	0.11	17.27***	6.69	97,423(16%)	1487(25%)
	60 to 69	1.56	0.11	13.68***	4.76	82,917(14%)	945(16%)
	70 to 79	1.40	0.12	11.57***	4.06	53,098(9%)	533(9%)
	80 or older	1.13	0.13	8.83***	3.10	32,585(5%)	317(5%)
Gender	Female	0	Fixed	Fixed	1	305,867(51%)	1887(32%)
	Male	0.96	0.03	30.00***	2.60	296,403(49%)	3967(68%)
Personal income/year	Less than 10,000	0	Fixed	Fixed	1	170,265(28%)	963(16%)
	10,000 to 20,000	−0.12	0.05	−2.42*	0.89	133,646(22%)	1878(32%)
	20,000 to 30,000	−0.21	0.06	−3.63***	0.81	96,273(16%)	1120(19%)
	30,000 to 40,000	−0.17	0.07	−2.49*	0.85	75,794(13%)	816(14%)
	40,000 to 50,000	−0.31	0.08	−3.89***	0.73	48,697(8%)	426(7%)
	50,000 to 75,000	−0.31	0.09	−3.52***	0.74	51,114(8%)	431(7%)
	75,000 to 100,000	−0.27	0.12	−2.23*	0.76	14,750(2%)	124(2%)
	100,000 to 150,000	−0.27	0.15	−1.72	0.77	8003(1%)	66(1%)
	More than 150,000	−0.44	0.22	−1.97*	0.64	3728(1%)	30(1%)
Household income/year	Less than 20,000	0	Fixed	Fixed	1	52,404(9%)	1147(20%)
	20,000 to 40,000	−0.18	0.05	−3.49***	0.84	129,459(21%)	1712(29%)
	40,000 to 60,000	−0.31	0.07	−4.65***	0.74	105,090(17%)	958(16%)
	60,000 to 80,000	−0.31	0.08	−4.11***	0.73	95,590(16%)	711(12%)
	80,000 to 100,000	−0.32	0.08	−3.77***	0.73	75,645(13%)	508(9%)
	100,000 to 150,000	−0.43	0.09	−4.85***	0.65	98,135(16%)	557(10%)
	150,000 to 200,000	−0.46	0.12	−3.86***	0.63	28,459(5%)	151(3%)
	More than 200,000	−0.26	0.14	−1.88	0.77	17,488(3%)	110(2%)
Household wealth	Less than − 100,000	0.09	0.11	0.84	1.10	13,279(2%)	98(2%)
	−100,000 to − 80,000	0.45	0.13	3.38***	1.57	6008(1%)	63(1%)
	−80,000 to −60,000	0.09	0.12	0.72	1.09	10,561(2%)	77(1%)
	−60,000 to −40,000	−0.00	0.09	−0.01	1.00	18,992(3%)	135(2%)
	−40,000 to −20,000	−0.08	0.08	−1.08	0.92	29,613(5%)	204(3%)
	−20,000 to 0	−0.01	0.05	−0.15	0.99	65,468(11%)	709(12%)
	0 to 20,000	0	Fixed	Fixed	1	132,799(22%)	1753(30%)
	20,000 to 40,000	0.05	0.06	0.84	1.05	38,994(6%)	356(6%)
	40,000 to 60,000	−0.03	0.08	−0.33	0.98	26,999(4%)	213(4%)
	60,000 to 80,000	0.06	0.08	0.69	1.06	21,684(4%)	184(3%)
	80,000 to 100,000	0.12	0.08	1.37	1.12	19,393(3%)	170(3%)
	100,000 to 150,000	0.08	0.06	1.31	1.09	43,924(7%)	362(6%)
	150,000 to 200,000	0.05	0.07	0.72	1.05	36,455(6%)	289(5%)
	More than 200,000	0.25	0.05	5.45***	1.29	138,101(23%)	1241(21%)
Education level	Unknown	0	Fixed	Fixed	1	243,871(40%)	2622(45%)
	Low	−0.04	0.05	−0.96	0.96	135,166(22%)	1138(19%)
	Middle	−0.05	0.04	−1.31	0.95	126,604(21%)	1338(23%)
	High	−0.20	0.05	−4.28***	0.82	96,629(16%)	756(13%)
Immigration background	Dutch	0	Fixed	Fixed	1	479,538(80%)	4861(83%)
	Western non-Dutch	0.15	0.04	4.23***	1.17	55,507(9%)	618(11%)
	Non-Western	−0.47	0.05	−10.28***	0.63	67,225(11%)	375(6%)
	1st generation immigrant	−0.33	0.04	−8.42***	0.72	66,276(11%)	490(8%)
	2nd generation immigrant	0.02	0.04	0.37	1.02	56,456(9%)	503(9%)
Urbanicity	Less than 10,000 people	0.16	0.15	1.05	1.17	5125(1%)	52(1%)
	10,000 to 100,000 people	0.10	0.04	2.67**	1.10	259,489(43%)	2465(42%)
	More than 100,000 people	0	Fixed	Fixed	1	332,825(55%)	3285(56%)
	Low address density	−0.00	0.04	−0.04	1.00	175,387(29%)	1689(29%)
	Medium address density	−0.00	0.04	−0.18	0.99	100,540(17%)	974(17%)
	High address density	0	Fixed	Fixed	1	321,512(53%)	3139(54%)
Place in household	Kid living at home	0	Fixed	Fixed	1	111,592(19%)	464(8%)
	Living alone	0.56	0.08	7.15***	1.75	114,055(19%)	2592(44%)
	Part non married couple without kids	−0.01	0.09	−0.14	0.99	41,826(7%)	378(6%)
	Part non married couple with kids	−0.46	0.10	−4.47***	0.63	132,559(22%)	990(17%)
	Part married couple without kids	−0.45	0.08	−5.66***	0.64	32,483(5%)	192(3%)
	Part married couple with kids	−0.55	0.08	−6.82***	0.58	128,513(21%)	768(13%)
	Member institutional household	0.02	0.11	0.18	1.02	10,339(2%)	183(3%)
	Parent of single parent household	−0.10	0.11	− 0.95	0.91	22,115(4%)	211(4%)
	Reference person other household	0.16	0.26	0.61	1.17	1578(0%)	17(0%)
	Other place household	−0.07	0.15	−0.45	0.94	7210(1%)	59(1%)
Healthcare costs/year	Less than 1000	0	Fixed	Fixed	1	407,142(68%)	2834(48%)
(excl. Mental health care)	1000 to 5000	0.33	0.03	9.85***	1.39	137,936(23%)	1816(31%)
	5000 to 10,000	0.59	0.05	11.41***	1.80	29,758(5%)	535(9%)
	More than 10,000	0.80	0.05	16.56***	2.23	27,434(5%)	669(11%)
Social benefits	Unfit for work benefits (UFW)	0.64	0.04	14.51***	1.89	25,196(4%)	966(17%)
	Long term unemployment benefits (LTU)	0.23	0.06	3.94***	1.26	22,089(4%)	577(10%)
	Short term unemployment benefits	0.19	0.06	3.33***	1.21	31,920(5%)	406(7%)
	Both UFW and LTU	−0.30	0.20	−1.51	0.74	699(0%)	32(1%)
Province	Groningen	0	Fixed	Fixed	1	17,791(3%)	232(4%)
	Drenthe	−0.06	0.10	−0.63	0.94	17,727(3%)	194(3%)
	Utrecht	−0.21	0.08	−2.52*	0.81	44,708(7%)	388(7%)
	Noord-Holland	−0.23	0.07	−3.13**	0.79	98,542(16%)	902(15%)
	Zuid-Holland	−0.21	0.07	−2.94**	0.81	127,000(21%)	1095(19%)
	Noord-Brabant	−0.01	0.07	−0.17	0.99	89,342(15%)	978(17%)
	Limburg	−0.21	0.08	−2.61**	0.81	40,603(7%)	409(7%)
	Overijssel	0.23	0.08	2.72**	1.26	40,315(7%)	354(6%)
	Flevoland	−0.11	0.12	−0.92	0.90	13,721(2%)	116(2%)
	Zeeland	−0.11	0.11	−0.98	0.90	13,449(2%)	141(2%)
	Gelderland	−0.09	0.07	−1.19	0.92	72,524(12%)	725(12%)
	Friesland	0.03	0.09	0.34	1.03	21,717(4%)	268(5%)
Year	2014	0	Fixed	Fixed	1	149,977(25%)	1406(24%)
	2015	0.10	0.04	2.59**	1.10	150,595(25%)	1468(25%)
	2016	0.10	0.04	2.62**	1.11	151,830(25%)	1475(25%)
	2017	0.14	0.04	3.70***	1.15	149,868(25%)	1505(26%)
Other	Self Employed	−0.05	0.06	−0.83	0.95	34,287(6%)	335(6%)
	Main Earner of Household	−0.06	0.05	−1.29	0.94	310,036(51%)	4244(72%)
	Has a legal debt repayment plan	−0.30	0.19	−1.58	0.74	1428(0%)	31(1%)
	In mental health care	2.04	0.03	64.97***	7.69	32,921(5%)	2134(36%)

Significance levels: *** < 0.001 < ** < 0.01 < * < 0.05

Taking the effect of possible correlating risk factors into account, significant increases in risk in all age groups were observed compared to those aged 10 to 19. We see large increases in particular among people aged between 40 and 49 (OR 5.70, 95% CI [4.57,7.24]), between 50 and 59 (OR 6.69, 95% CI [5.37,8.33]), and between 60 and 69 (OR 4.76, 95% CI [3.82,5.93]).

The fact that males die more often due to suicide than females (OR 2.60, 95% CI [2.46,2.77]) still holds when corrected for other characteristics. Furthermore, having mental health problems (OR 7.69, 95% CI [7.24,8.17]) as well as physical health problems as measured through healthcare costs (up to OR 2.23, 95% CI [2.01,2.46]) are major risk factors. Additionally, living alone (OR 1.75, 95% CI [1.49,2.05]), and all forms of unemployment, especially those that have been found unfit for work (UFW; having an OR of 1.89, 95% CI [1.75,2.05]), increase the risk of suicide.

Looking at protective factors, the analyses show that people with a high level of education have a low risk (OR 0.82, 95% CI [0.74,0.90]). Low-risk people are also those with a non-western immigration background (OR 0.63, 95% CI [0.57,0.69]) and 1st generation immigrants (OR 0.72, 95% CI [0.66,0.78]). Also being married or having children is a protective factor for a couple living together (OR 0.64, CI 95% [0.54.0.75] for a married couple without kids, OR 0.63, 95% CI [0.52,0.77] for a non-married couple with kids). These effects are weaker when the other effect is already present (OR 0.58, 95% CI [0.48,0.69]).

Having a higher income is also a protective factor. This holds for both personal income (up to OR 0.64, 95% CI [0.41,1]) as well as household income (up to OR 0.63, 95% CI [0.50,0.80]). Interestingly, household wealth does not appear to be a protective factor. It even increases risk in the wealthiest category (Table 1). We observe urbanicity and regional differences being mostly non-significant.

**Fig. 1:**
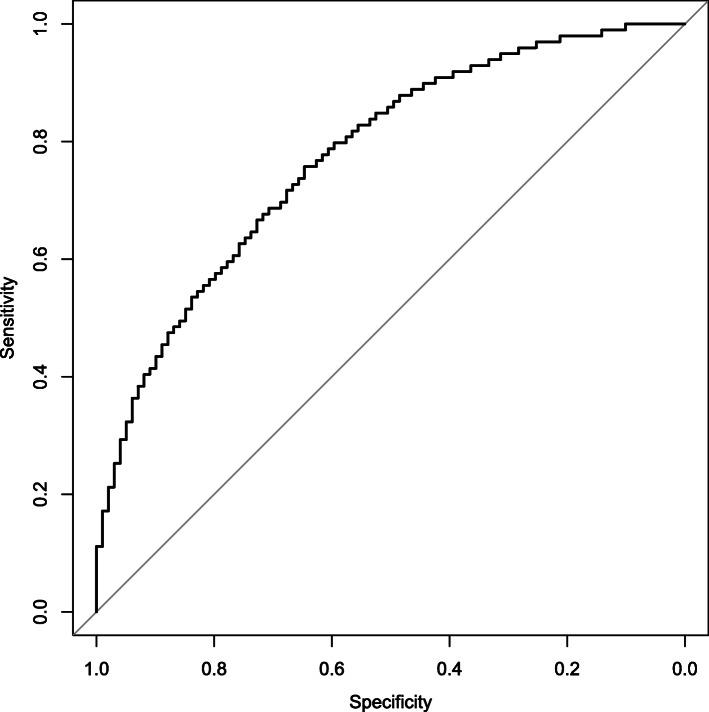
Sensitivity-specificity plot of model predictions. The area under the curve is 0.77

Figure 1 shows the approximate ROC (based on percentiles to preserve privacy). Each point on the curve corresponds to a threshold and shows the proportion of people who died by suicide that are above the threshold (the sensitivity) on the y-axis. On the x-axis, it shows the proportion of people in the control group who are above the threshold. The curve shows a trade-off between true and false positives and allows for an informed choice of thresholds for risk groups. The AUC, which is based on the full plot, is 0.77. This means that the probability that an individual in the sample of those dying by suicide will get a higher predicted risk than an individual in the control set is 77%. A fully random model would have an AUC of 0.50, while a perfect model would have an AUC of 1.

## Discussion

To our knowledge, this is the first study done into suicide on socio-demographic factors with such a large and unbiased sample, where, due to the level of detail of the data, analyses could be done to control for many characteristics, giving us very robust risk factors. We found that previously discovered risk factors for suicide (middle-age, male gender, and unemployment (as measured through benefits)) remain elevated even when corrected for a wide array of socio-demographic characteristics. The same holds for commonly found protective factors for suicide, like having a higher income or immigration background.

Most increased risk came from being a recipient of mental health care (which includes being an inpatient as well as being an outpatient), which can be expected knowing that approximately 87% of people who die by suicide have mental health problems [10]. Additionally, physical healthcare being a risk factor could be explained due to hospitalisations for previous suicide attempts. However, due to the fact that the risk keeps increasing as physical health care costs increase, it is unlikely this would account for all of the increased risk.

This study did not observe significant differences between rural and urban municipalities. However, it is important to note that due to the high population density in the Netherlands, most rural areas in the Netherlands might still be considered urban compared to rural areas in other countries.

Looking at raw frequencies, we see regional differences in the Netherlands [11]. These differences became much less when the effects of possible correlating risk factors were considered. This seems to indicate that the regional differences are primarily caused by the differences in the demographic makeup of the regions as opposed to specific local causes.

When we look at level of education, we see that being highly educated remains a protective factor. However, this only holds for the highest level of education and is not particularly protective. Especially when compared to the results of Phillips and Hempstead [12] who found large differences between the suicide rates among people with a high school degree and those among people with a college degree in the United States. Combined with the protective factor of income and the high correlation between level of education and income, this seems to suggest a proxy effect. The level of education might only be a protective factor due to the associated increase in income.

Our model has a reasonable fit with an AUC of 0.77, which is high for a model predicting suicide death [1] and comparable to the recent results of Zheng et al. [13] who used deep neural networks on electronic health records to predict suicide attempts (AUC of 0.769). It could be used to identify low, regular, or high-risk groups. However, the model is not usable to predict suicide risk in individuals. Suicide is a rare event that on average occurs in about 1 in 10,000 people a year. This means that even if you have a tenfold increase in predicted risk, you will still have 1000 false positives for each true positive.

Although then not useful for prediction on an individual level, the results from this study allow for targeted prevention measures at certain risk groups. For example, it would be possible to train social workers that are in regular contact with recipients of social benefits to be gatekeepers. Alternatively, high risk groups may be specifically targeted to raise awareness of suicide prevention hotlines within these groups. The authors also recommend that this study is repeated at regular intervals to see whether changes in public policy coincide with changes in risk groups.

The methodology used in this study allowed us to find robust risk and protective factors for suicide. However, with this methodology it is not possible to discover which specific combinations of risk factors or protective factors are especially dangerous or safe. Research has shown that the interactions of risk factors play a substantial role in suicide prediction and greatly improves model performance [13]. Therefore, having a proper understanding of such interactions will be of great importance in future research. We are currently working on a new machine learning model that will allow us to find significant interactions in a data-driven and hypothesis-free manner. Since we are doing this in a data driven and hypothesis-free manner, it both limits bias on which interactions to include and allows us to discover interactions that have not even been considered before.

## Data Availability

Due to privacy reasons the data used can only be accessed on the servers of Statistics Netherlands for which researchers can request access. Access is granted at the discretion of Statistics Netherlands.
